# Photo‐Responsive Room‐Temperature Phosphorescent Films Exhibiting Photochromism and Time‐Dependent Phosphorescent Colors for Multilevel Anti‐Counterfeiting

**DOI:** 10.1002/advs.202523121

**Published:** 2026-03-03

**Authors:** Ziyi Bai, Xinyi Xu, Jinbo Fu, Di Zhao, Yige Wang, Huanrong Li

**Affiliations:** ^1^ School of Chemical Engineering and Technology Hebei University of Technology Tianjin P. R. China

**Keywords:** anti‐counterfeiting, color‐tunability, photoresponse, time‐dependent phosphorescence colors

## Abstract

Developing dynamic color‐tunable room‐temperature phosphorescence (RTP) materials with photochromism and time‐dependent phosphorescence color (TDPC) presents a promising but highly challenging approach for achieving multilevel anti‐counterfeiting applications. Herein, we demonstrate a facile in situ synthesis strategy to photo‐responsive color‐tunable RTP polymer films with reversible photochromic and TDPC properties, which was achieved through rational integration of carboxyl‐functionalized spiropyran (SPCOOH) and luminescent hydrogen‐bonded organic framework formed by melamine (ME) and isophthalic acid (IPA) within the PVA amorphous matrix, mediated by synergistic hydrogen‐bonding networks. Crucially, UV‐induced spiropyran‐to‐merocyanine (SP→MC) isomerization enables a novel triplet‐singlet Förster resonance energy transfer (TS‐FRET) pathway between the ME‐IPA donor and MC acceptor, achieving precise modulation in afterglow color from cyan to orange. Moreover, the energy transfer creates distinct decay kinetics, thereby endowing the material with TDPC property. Furthermore, their multilevel time‐resolved multicolor dynamic anti‐counterfeiting applications have been demonstrated. This work establishes a simple and effective strategy for constructing TDPC systems to develop color‐tunable RTP materials.

## Graphical abstract

A series of photo‐responsive polymers are prepared by a facile one‐pot in situ synthesis strategy, where RTP modules and photochromic units are systematically integrated into one polymer system. The triplet‐singlet Förster resonance energy transfer (TS‐FRET) between the donor and acceptor enables the material to exhibit time‐dependent phosphorescence with multicolor tunability.

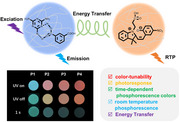

## 1 Introduction

Room‐temperature phosphorescent (RTP) materials have important applications in the fields of bio‐imaging, anti‐counterfeiting and information security owing to their long‐lived emissions [1, 2, 3, 4, 5, 6, 7, 8]. Currently, most of RTP materials are rare‐earth and/or transition metal‐containing inorganics, suffering from high cost, high toxicity and harsh preparation conditions [3, 6, 7]. The organic RTP materials have been attracting great attention because of their lower cost and toxicity compared with the inorganic counterparts [1, 9, 10, 11]. The researchers have developed a versatile of organic RTP materials by enhancing intersystem crossing (ISC) efficiency through molecular structure optimization to boost triplet exciton generation and constructing suitable molecular environments to suppress non‐radiative triplet‐state decay [12, 13, 14, 15, 16, 17, 18]. Although notable progress has been made in color‐tunable RTP materials, most systems exhibit static phosphorescence colors once fabricated, and few integrate reversible photochromism with time‐dependent afterglow color evolution. For instance, recent works have focused on host‐guest doping for color‐tunable RTP [19, 20] or flexible RTP polymers with fixed afterglow hues [21, 22]. The development of RTP materials that combine photochromism, dynamic afterglow color, and photo‐switchable energy transfer remains a significant challenge.

In contrast, the materials with time‐dependent phosphorescent color (TDPC) capabilities can provide additional channels in the time dimension, rendering them particularly suitable for multidimensional encryption and advanced anti‐counterfeiting applications [23, 24, 25, 26, 27, 28]. The successful realization of TDPC is largely relying on the design of multiple emissive centers with differentiated but comparable lifetime via various strategies such as structure defect engineering, aggregated phosphorescence, multi‐component doping and the non‐radiative energy transfer between delayed fluorescence components and RTP components. For instance, Yang et al. observed TDPC in an organic molecular crystals, ascribed to the well‐separated dual emissions of long‐persisted thermally activated delayed fluorescence and RTP with comparable but different lifetimes [25]. Feng and co‐workers achieved TDPC in Xylan‐carbonized polymer dots via the confinement‐modulated clusterization strategy [28]. Hirata et al. developed a TDPC material through tuning the aggregation of the amino‐substituted aromatic compounds in a hydroxyl steroid host matrix [29]. Zong et al. [30]. incorporated Mn^2^^+^ ions into aluminophosphate zeolite AlPO‐5 by a host matrix control strategy. This doping creates distinct microenvironments for embedded carbon dots (CDs), enabling temperature‐controlled multimodal TDPC. However, the development of TDPC materials is still at the preliminary stage, and most of the reported systems face significant challenges, including complex multi‐step synthesis processes, excessively high production costs, and insufficient light stability, which collectively hinder their practical implementation. Moreover, most of the reported TDPC materials lack photochromism, i.e., the appearance color of the materials changed distinctly under light illumination owing to the chemical structure conversions of special molecules triggered by light [31, 32, 33, 34, 35]. Endowing the TDPC materials with the fascinating photochromic property might open up new gateways for innovation because the TDPC phenomena and material color can be manipulated by light, thus empowering the materials with a multilevel encryption mode. Although there are currently no reports on these aspects, we envision that such advancements will unlock exciting possibilities in material science. Therefore, developing a facile way to photochromic TDPC materials is of great significance and highly desirable. However, this remains a great challengeable task since the rigid environment required by the efficient RTP can deactivate the photochromism via restricting the conformational changes of the molecules during isomerization caused by photo‐irradiation [20, 36, 37].

Herein, we propose a facile strategy for achieving photochromic films with TDPC property by simply doping RTP modules and the photochromic units within a polymer matrix. This approach enables the systematic integration of various functional components, yielding a series of polymer films that exhibit tunable TDPC behavior under light stimulation. Specifically, a luminescent hydrogen‐bonded organic framework formed by melamine (ME) and isophthalic acid (IPA), denoted as ME‐IPA, was selected as the phosphorescent component for constructing multicolor‐tunable phosphorescent polymers; Carboxyl‐functionalized spiropyran (SPCOOH) was incorporated to impart photochromism and photo‐responsive properties, while polyvinyl alcohol (PVA)—rich in hydroxyl groups enabling extensive hydrogen‐bonding—served as the polymer matrix for inhibiting the molecular motion of the guest. Through this design, the color‐tunable RTP polymer films with TDPC characteristics and photochromic properties were successfully fabricated. Under 365 nm UV irradiation, a triplet‐singlet Förster resonance energy transfer (TS‐FRET) process is activated between the triplet state of ME‐IPA (donor) and the singlet state of its photoisomerized merocyanine form (MC, acceptor). This energy transfer suppresses the green phosphorescence of the donor at 493 nm and enhances the red phosphorescence of the acceptor at 655 nm, resulting in distinguishable changes in both phosphorescent color and daylight visual appearance with an interesting TDPC phenomenon, manifesting a distinguishable and dynamic afterglow color evolution from light orange to green. Furthermore, the film exhibits full photochemical reversibility: irradiation with visible light (> 405 nm) triggers the reconversion from MC back to SP state, thereby terminating the TS‐FRET process and restoring the original luminescent state. Additionally, we designed intricate security labels with multilevel encryption capabilities, demonstrating a novel strategy for advanced multidimensional anti‐counterfeiting applications. Compared to earlier RTP systems that often rely on static emission or complex multi‐step synthesis, our strategy offers a facile one‐pot route to films with reversible photochromism and TDPC. The unique TS‐FRET mechanism enables precise light‐controlled afterglow color modulation, providing multi‐level encryption capabilities that surpass conventional single‐channel anti‐counterfeiting technologies. Given the growing demand for processable and dynamic luminescent materials in flexible electronics and advanced security [38, 39], this work opens a promising avenue for next‐generation smart optical materials.

## 2 Results and Discussion

### 2.1 Design, Synthesis, and Characterization

**SCHEME 1:**
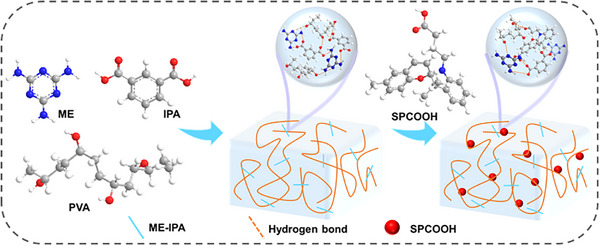
Probable chemical structures of **ME‐IPA‐SP_n_@PVA**.

In order to develop the photochromic and color‐tunable RTP polymers featuring TDPC properties, PVA polymer was selected as the rigid matrix to host the phosphorescent ME‐IPA [10, 40, 41] and photochromic block SPCOOH [42] for it can provide a rigid microenvironment to suppress the non‐radiative relaxation, thereby generating efficient RTP [43]. Considering the poor aqueous solubility of ME‐IPA, we adopted an in situ synthesis strategy by adding IPA into the aqueous solution of ME and PVA, enabling uniform generation of ME‐IPA within the PVA network, denoted as ME‐IPA@PVA. The subsequently introduced SPCOOH also binds efficiently to the PVA network owing to the network's abundant free hydroxyl groups that provide numerous hydrogen‐bonding sites, enabling both ME‐IPA and SPCOOH to readily anchor via hydrogen‐bonding. Thus, a series of photochromic and color‐tunable RTP polymers featuring TDPC characteristics designated as ME‐IPA‐SP_n_@PVA (*n* = 0, 0.01, 0.04, 0.07) were obtained through precise modulation of the ME‐IPA/SPCOOH ratio. The chemical structures of ME‐IPA‐SP_n_@PVA is illustrated in Scheme 1. Due to its superior optical properties (discussed in detail later), ME‐IPA‐SP_0.04_@PVA was selected as the representative sample for further investigation.

**FIGURE 1:**
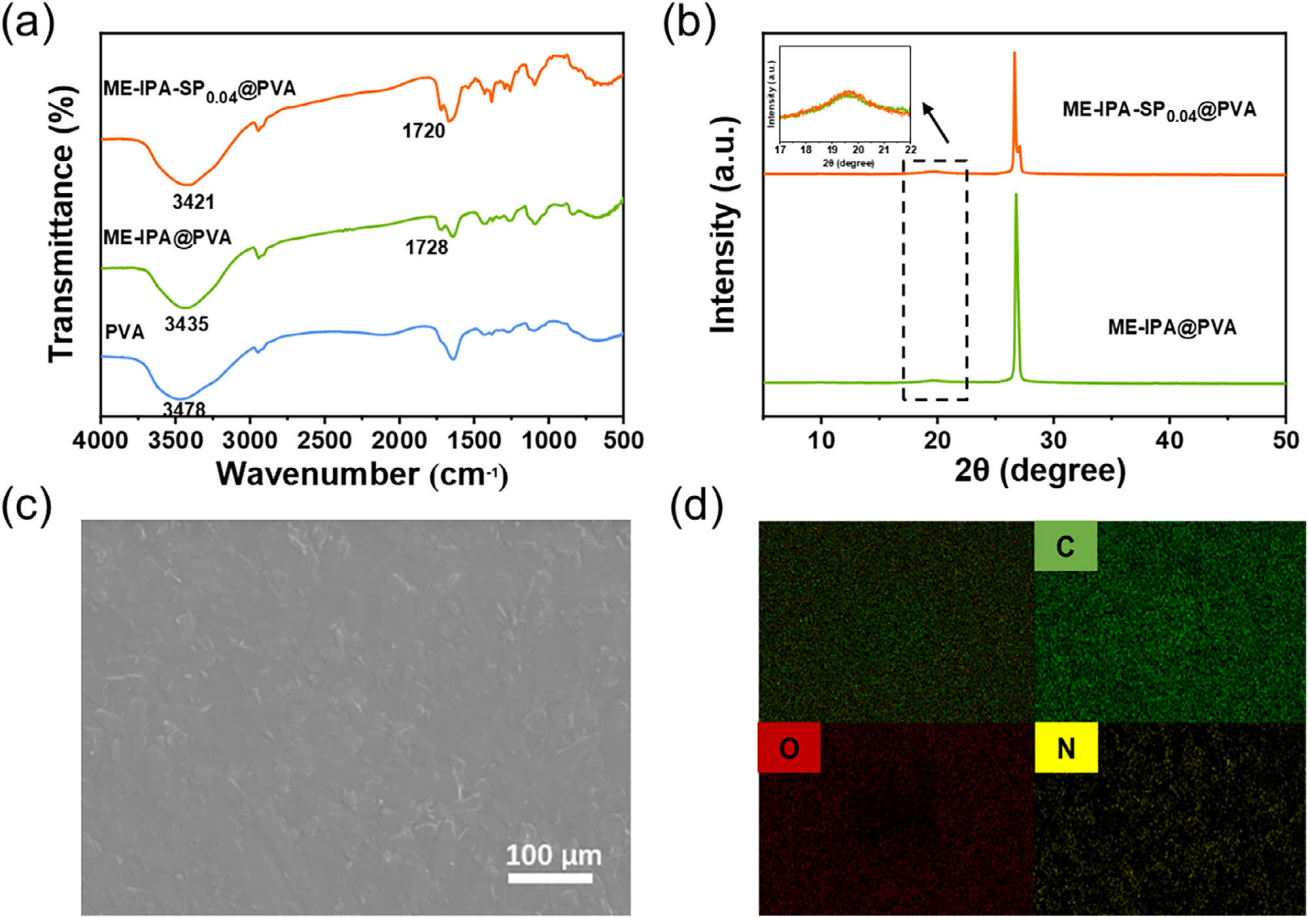
(a) FT‐IR spectra of PVA, **ME‐IPA@PVA** and **ME‐IPA‐SP_0.04_@PVA** films; (b) XRD spectra of **ME‐IPA@PVA** and **ME‐IPA‐SP_0.04_@PVA** films; (c) SEM surface morphology of **ME‐IPA‐SP_0.04_@PVA** film; (d) EDX mapping of **ME‐IPA‐SP_0.04_@PVA** film.

The entire synthesis process of ME‐IPA‐SP_0.04_@PVA was monitored by FT‐IR spectrum (Figure 1a). Upon incorporation of ME‐IPA and SPCOOH, the ‐OH stretching vibration band observed in the FT‐IR spectrum of PVA gradually shifted from 3478 cm^−^^1^ for to 3421 cm^−1^, indicative of hydrogen bond formation (O─H···O and O─H···N) between the polymer matrix and the functional groups of the dopants [44]. Additionally, the C═O stretching vibration band at 1720 cm^−^^1^ in ME‐IPA‐SP_0.04_@PVA showed a redshift compared to that of ME‐IPA@PVA, further confirming hydrogen‐bonding interactions. XRD patterns of the polymer films display a strong diffraction peak at 26.6°(Figure 1b), with the interplanar spacing of 3.35 Å calculated from the Bragg equation, confirms that the ME‐IPA molecules are tightly packed to form a layered supramolecular structure [40, 41]. This rigid lamellar stacking effectively suppresses non‐radiative transitions, thereby establishing the structural foundation for the material's ultralong RTP [41]. Furthermore, a broad diffraction peak at 19.6° corresponding to the amorphous PVA matrix with randomly coiled polymer chains was also observed [44]. Importantly, no significant shift in the diffraction peaks was observed in ME‐IPA‐SP_0.04_@PVA compared to ME‐IPA@PVA, demonstrating that the introduction of the SPCOOH component did not compromise the lattice integrity of ME‐IPA. These results collectively confirm the successful synthesis of ME‐IPA‐SP_0.04_@PVA films. SEM image of ME‐IPA exhibited a bulk crystal structure (Figure S1). In the ME‐IPA@PVA film, ME‐IPA is homogeneously dispersed within the PVA network (Figure 1c). Simultaneously, EDX mapping demonstrated uniform distribution of nitrogen (N) elements in ME‐IPA‐SP_0.04_@PVA (Figure 1d), further confirming the effective dispersion of both ME‐IPA and SPCOOH within the PVA matrix.

### 2.2 Photochromic Properties

*η*_ET_ represents the energy transfer efficiency between ME‐IPA and MC, which is calculated by the following Equation (1): [45]

$$\eta_{\mathrm{ET}}=1-\frac{\tau }{\tau_{0}}$$

(1) $$\eta_{ET}=1-\;\tau /\;\tau_{0}$$

where *τ* and *τ_0_* represent the lifetimes of **ME‐IPA‐SP_n_@PVA** and **ME‐IPA@PVA** films, respectively

**FIGURE 2:**
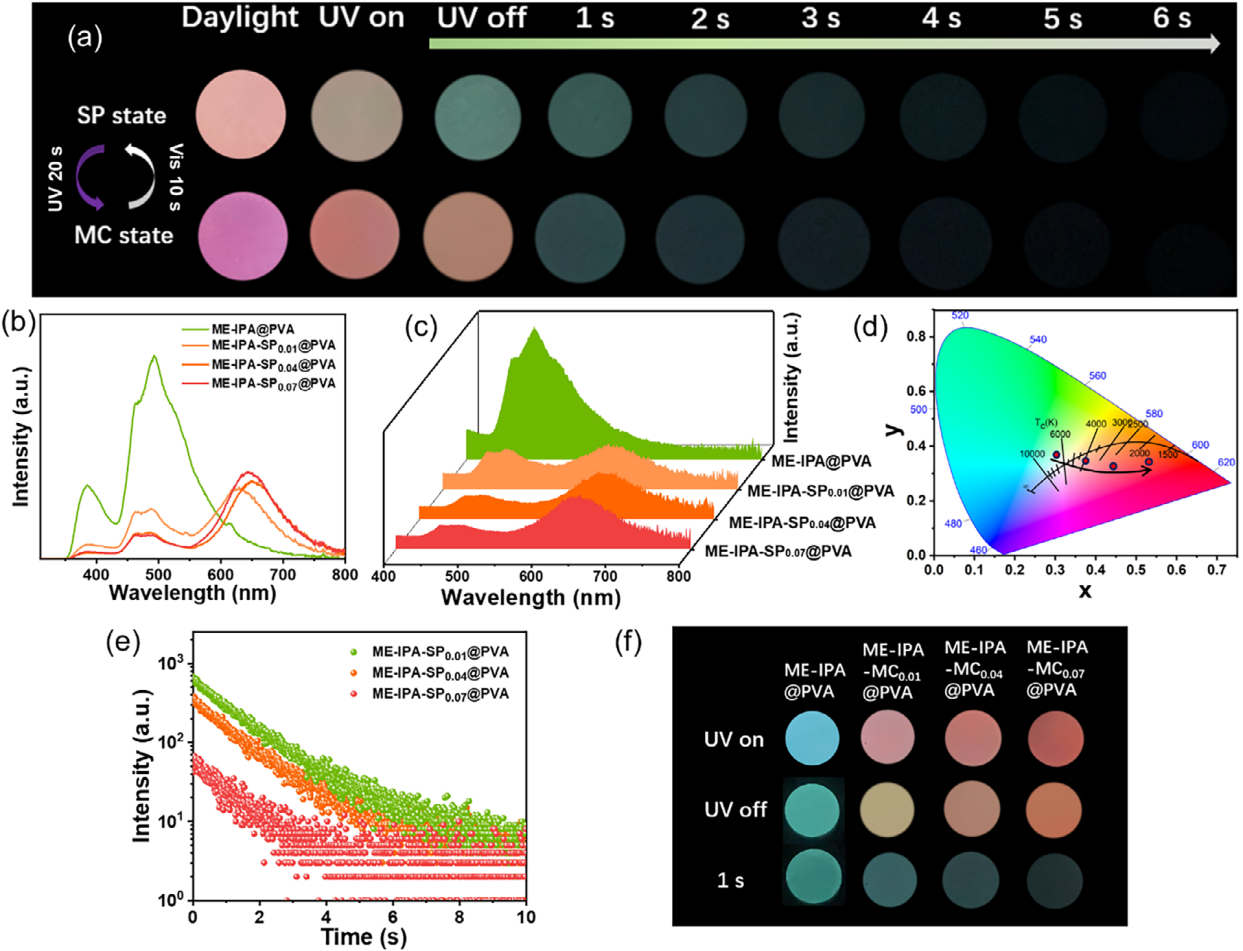
Luminescence properties of **ME‐IPA‐SP_n_@PVA** films. (a) Photographs of **ME‐IPA‐SP_0.04_@PVA** (SP and MC state, which controlled by the UV light of 365 nm and visible light of >405 nm) upon daylight, 302 nm UV on and UV off conditions; (b) Fluorescence emission spectra (λ_ex_ = 300 nm, same slit mode); (c) Phosphorescence emission spectra (λ_ex_ = 300 nm, same slit mode, with an interval time of 0.5 ms(applicable to all phosphorescence spectra)); (d) CIE afterglow emission coordinates; (e) Phosphorescence decay curves (λ_ex_ = 300 nm, λ_em_ = 493 nm); (f) Photographs of luminescence behaviors upon 302 nm UV on and UV off.

Under UV‐light irradiation, all the synthesized polymers showed variable emission ranging from blue to red depending on the SPCOOH content (Figure 2a; Figures S4 and S5). Moreover, all the samples exhibited bright ultralong color‐variable afterglow after ceasing the UV‐light source, with that of **ME‐IPA‐SP_0.01_@PVA** (MC state, after UV irradiation) persisting for 6 s observed by the naked eyes (Figure 2a; Figures S4 and S5). Their steady‐state photoluminescence (PL) and phosphorescence spectra are shown in Figure 2. The peaks observed at 385 nm in PL spectra of all samples are ascribed to the fluorescence emission of the ME‐IPA units entrapped within the PVA matrix (Figures S6 and S7). The introduction of the SPCOOH resulted in the appearance of an emission peak at above 620 nm derived from the S_1_ to S_0_ transition of the sipropyran moieties in the polymer [33]. The green emission bands peaking at about 493 nm and red emission bands peaking at above 620 nm are observed in both the PL and phosphorescence emission spectra (Figure 2b,c). The green afterglow was ascribed phosphorescence emission of the ME‐IPA units within the PVA matrix [40, 41]. Notably, these green and red emission bands exhibited inversely correlated intensity variations with increasing SPCOOH content. Figure 2d illustrates a green‐to‐orange afterglow color shift in the CIE diagram, correlating with visual observations (Figure 2f). Concomitantly, the lifetime of the green phosphorescence progressively decreased from 1.56 to 1.10 s, while that of red components increased from 0.70 to 0.95 s (Figure 2e, Table 1, and Figure S8). Noticeably, without ME‐IPA chromophores, **SP@PVA** in the MC state can only emit red photoluminescence under UV irradiation, and no detectable red phosphorescence was observed after ceasing the UV‐light source (Figures S9 and S10). Meanwhile, **ME‐IPA@PVA** exhibits only intense green afterglow persisting for longer than 10 s, and no red afterglow can be observed (Figure S9). These results strongly indicated that red afterglow emission can be ascribed to the triplet‐singlet Förster resonance energy transfer (TS‐FRET) between the triplet state of ME‐IPA and the singlet state of SPCOOH. The observed afterglow color variation arises from the UV‐triggered SP→MC isomerization, which activates TS‐FRET between ME‐IPA (donor) and MC (acceptor). This energy transfer quenches the green emission (493 nm) and enhances the red emission (655 nm), leading to an orange–red afterglow immediately after UV cessation. Therefore, the lifetime of SPCOOH in polymer was prolonged from nanoseconds to hundreds of millisecond scale (Figure 4b; Figure S11).

**TABLE 1:** Phosphorescence lifetime and TS‐FRET efficiency of different polymers.

Samples	Lifetime (464 nm)/s	Lifetime (493 nm)/s	Lifetime (625 nm)/s	Lifetime (655 nm)/s	*η_ET_* /%
ME‐IPA@PVA	1.34	1.56	/	/	/
ME‐IPA‐SP_0.01_@PVA	1.13	1.25	0.70	/	19.9
ME‐IPA‐SP_0.04_@PVA	1.11	1.23	/	0.95	21.2
ME‐IPA‐SP_0.07_@PVA	1.09	1.10	/	0.90	29.5

To elucidate the quantitative relationship between SP→MC photoisomerization and energy transfer efficiency, the UV‐induced conversion of SPCOOH to its MC form in ME‐IPA‐SP0.04@PVA films was monitored by UV–vis spectroscopy. Concurrently, phosphorescence decay curves were performed, and the corresponding energy‐transfer efficiency ηET was derived using Equation (1) (Table S1). The results indicate that the SP→MC conversion proceeded rapidly within the first 5 s, reaching a photostationary state after 10 s (Figure S12). Accordingly, ηET also reached its maximum value at 5 s of irradiation, slightly decreased at 10 s, and then remained basically unchanged (Figure S13). The saturation in ηET after 5 s indicates that nearly all SP units in the system had been isomerized to MC, and no further enhancement in energy transfer was achieved by continued irradiation.

**FIGURE 3:**
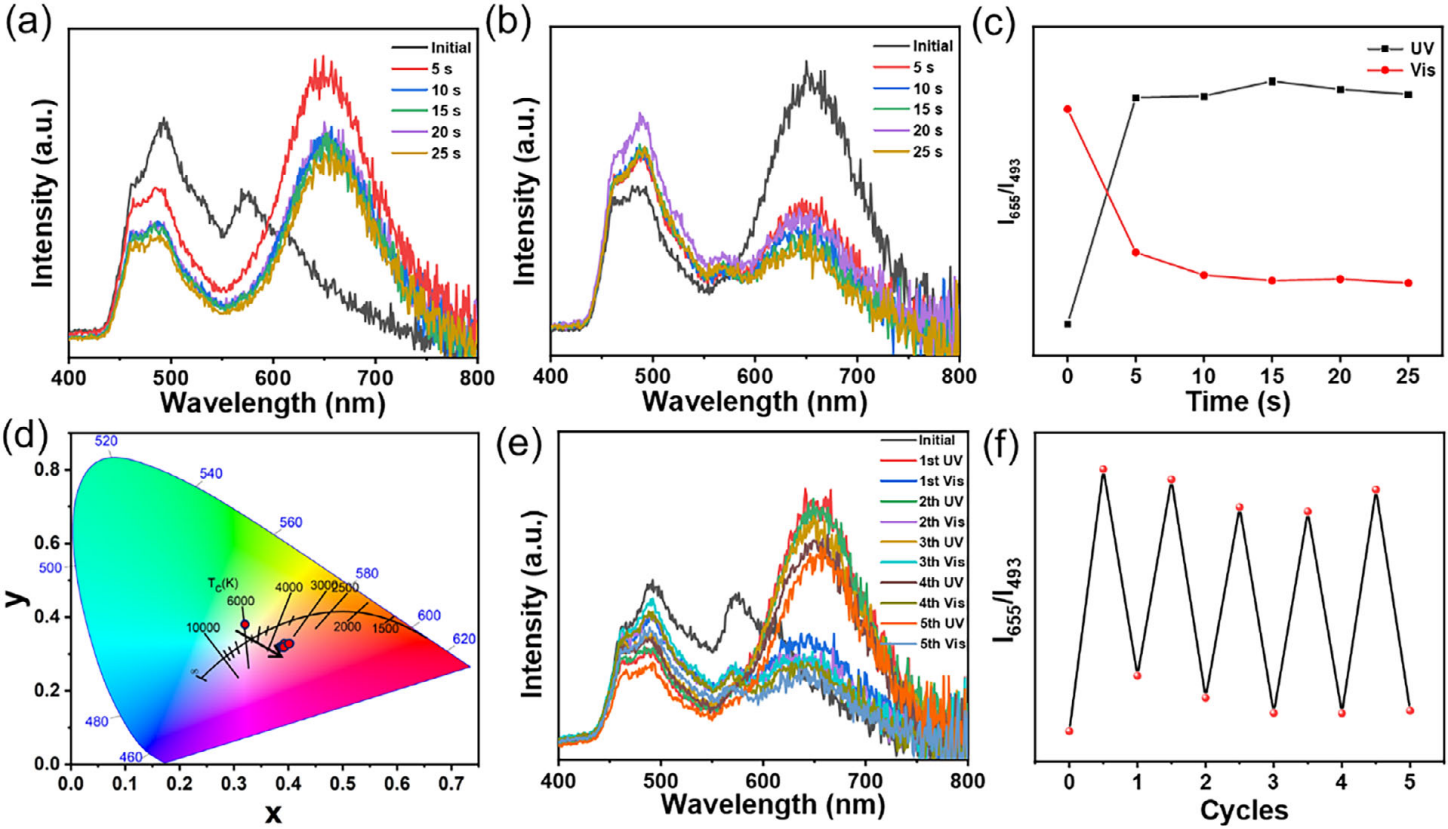
Time‐dependent phosphorescence spectra of **ME‐IPA‐SP_0.04_@PVA** film under (a) 365 nm UV irradiation and (b) visible light irradiation; (c) The phosphorescence proportional intensity changes at 655 and 493 nm during the UV and visible light irradiation; (d) CIE coordinates of afterglow emission under the 365 nm UV irradiation; (e) Phosphorescence emission spectrum of **ME‐IPA‐SP_0.04_@PVA** film under continuous alternating UV and visible irradiation (λ_ex_ = 300 nm); (f) The coloration/fading cycles of the **ME‐IPA SP_0.04_@PVA** film (for the UV irradiation time: 10 s; the visible light irradiation time: 20 s).

The light‐responsive feature of the SPCOOH component enables multicolor afterglow emission modulation not only through SPCOOH concentration variation but also via UV irradiation (Figure 2a). To investigate the photo‐stimulative responsive behavior, the **ME‐IPA‐SP_0.04_@PVA** sample was subjected to sufficient pre‐irradiation with visible light, ensuring that the conversion of the spiropyran to its closed‐ring SP form as completed as possible prior to testing. Subsequent continuous 365 nm UV irradiation induced a pronounced increase in the characteristic broad emission peak of the open‐ring merocyanine (MC) form centered at 655 nm, concomitant with a gradual decrease in the green emission peak of ME‐IPA at 493 nm in the time‐dependent phosphorescence spectra of **ME‐IPA‐SP_0.04_@PVA** (Figure 3a). These opposing intensity trends resulted in a distinct phosphorescence color shift from green to light orange within 10 s of UV exposure. The dynamic luminescence behavior is quantitatively illustrated in the CIE diagram (Figure 3d), where the coordinates migrated from (0.320, 0.383) to (0.405, 0.332). Meanwhile, this emission change was accompanied by a visual color transition of the film from light pink to purple (Figure 2a; Figures S2 and S3), ultimately reaching a photostationary state (Figure 3c). Moreover, **ME‐IPA‐SP_0.04_@PVA** exhibits excellent photoisomerization reversibility (Figure 3b,c), both emission intensity and color fully revert to initial states within 20 s of visible‐light irradiation (> 405 nm). Critically, the film maintains high photo‐switching fidelity through five consecutive UV/vis cycles (Figure 3e,f), demonstrating exceptional fatigue resistance. In addition, the photo‐controlled afterglow behaviors of **ME‐IPA‐SP_0.01_@PVA** (Figures S4 and S16) and **ME‐IPA‐SP_0.07_@PVA** (Figures S5 and S15) were monitored. The changes of SPCOOH content in these samples resulted in altered ranges of afterglow color change within their respective CIE diagrams. Collectively, these results confirm that the photo‐responsive RTP properties originate from the reversible photoisomerization between the SP and MC states of the SPCOOH component.

**FIGURE 4:**
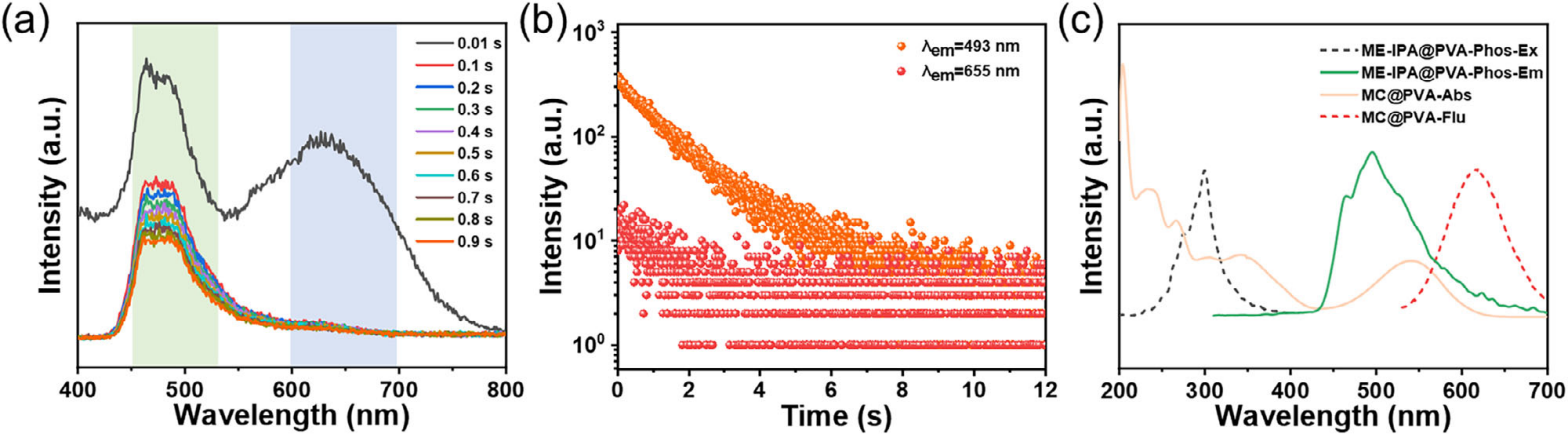
(a) Time‐resolved phosphorescence spectra of **ME‐IPA‐SP_0.04_@PVA** (MC state)(λ_ex_ = 300 nm); (b) RTP lifetime decay curves of **ME‐IPA‐SP_0.04_@PVA**, λ_ex_ = 300 nm, λ_em_ = 493 and 655 nm; (c) The phosphorescence spectra of **ME‐IPA@PVA** and the fluorescence/ UV–vis absorption spectrum of **MC@PVA** (λ_ex_ = 300 nm).

Notably, the unique TDPC phenomena can be triggered by UV light. The **ME‐IPA‐SP_0.04_@PVA** sample exposed to 365 nm for 10 s showed a phosphorescence color transition from light orange to green within 1.0 s after ceasing 302 nm excitation (Figure 2a), whereas neither **ME‐IPA@PVA** nor **SP@PVA** samples does not exhibit the TDPC phenomenon (Figure S6). Initially, the red emission intensity of **ME‐IPA‐SP_0.04_@PVA** was comparable to the green luminescence intensity. Subsequently, the red afterglow (lifetime of 0.95 s, 655 nm) decayed more rapidly, whereas the green afterglow (lifetime of 1.23 s, 493 nm) gradually became the dominant component (Figure 4a,b). This differential decay behavior resulted in a dynamic color evolution from light orange to green. To investigate the dynamic luminescence mechanism of **ME‐IPA‐SP_n_@PVA**, the phosphorescence spectra of **ME‐IPA@PVA** and fluorescence/UV–vis absorption spectra of **SP@PVA** exposed to 365 nm light for 10 s were investigated. As shown in Figure 4c, the broad absorption band of **SP@PVA** centered at 543 nm overlaps significantly with the phosphorescence emission peak of **ME‐IPA@PVA**, which provides the possibility for energy transfer in the **ME‐IPA‐SP_n_@PVA** system [46, 47, 48]. With the increase of the proportion of SPCOOH in the samples, the characteristic peak intensities and decay lifetimes of ME‐IPA at 493 nm gradually decreased, while that of SPCOOH were enhanced, and the energy transfer efficiency (*η*_ET_) was increased from 19.9% to 29.5% (Table 1). Furthermore, in the ME‐IPA‐SP_n_@PVA system, the phosphorescence emission peak of the acceptor does not exhibit any redshift compared to its fluorescence emission peak. Taking ME‐IPA‐SP_0.04_@PVA as an example, both peaks are located at 655 nm. This observation precludes the possibility of triplet–triplet energy transfer (TTET) as the operative mechanism (Figure 2b,c) [49, 50]. These results collectively demonstrate the occurrence of triplet‐to‐singlet Förster resonance energy transfer (TS‐FRET) from ME‐IPA donor to SPCOOH acceptor in the **ME‐IPA‐SP_n_@PVA**.

**FIGURE 5:**
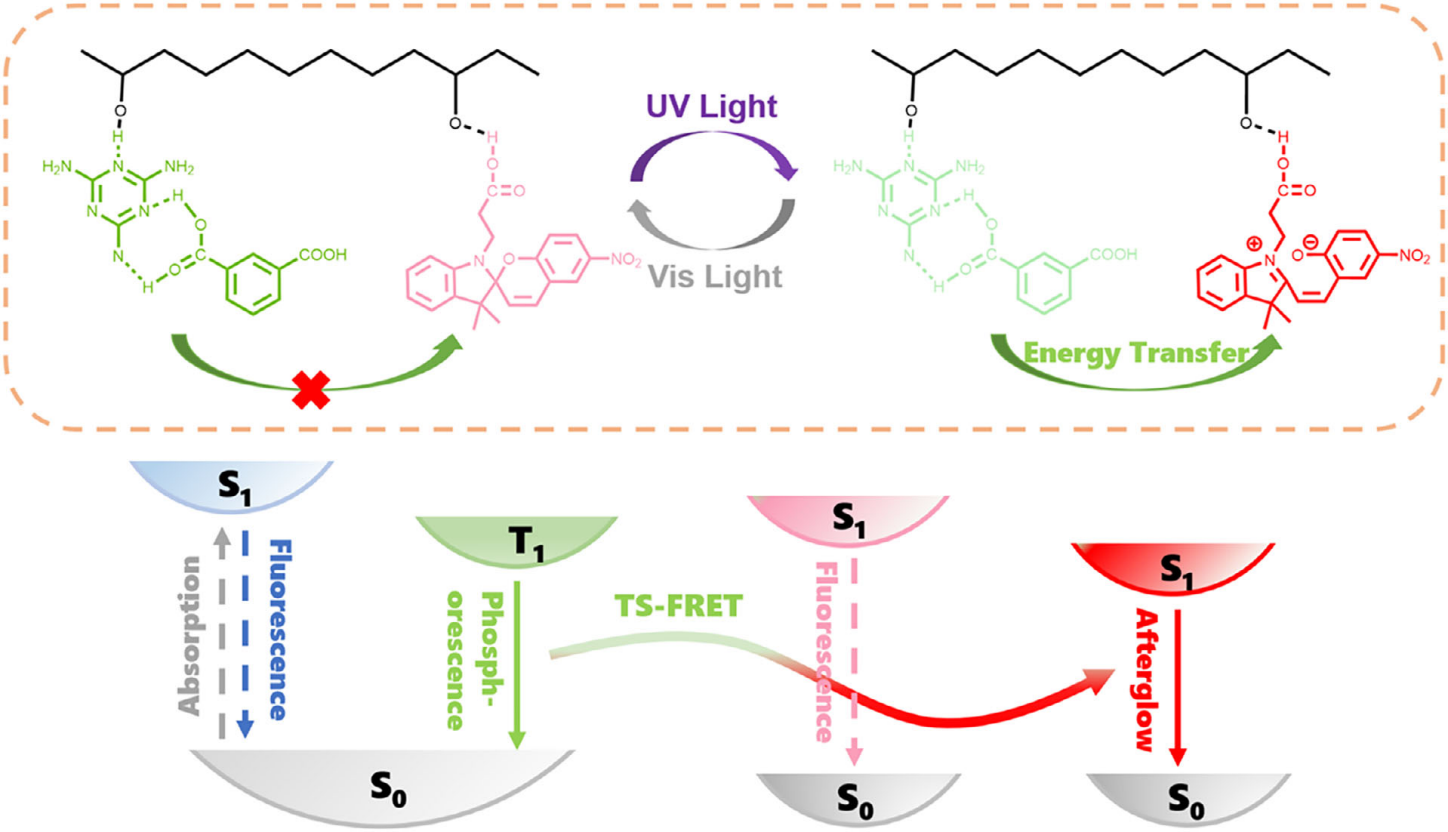
Mechanism and illustration of FRET processes in **ME‐IPA‐SP_n_@PVA** system.

On the basis of the above results, we proposed a dynamic luminescence mechanism for **ME‐IPA‐SP_n_@PVA** under external light stimulation (Figure 5). With the irradiation of UV light (l = 365 nm), SPCOOH molecules are isomerized from the closed‐ring form SP to the open‐ring form MC. At the same time, there is FRET from the ME‐IPA donor to the SPCOOH acceptor, resulting in the coexistence of dual phosphorescent centers (ME‐IPA and SPCOOH) in the system. Owing to their different lifetime and intensity, **ME‐IPA‐SP_n_@PVA** polymers acquire time‐dependent color‐changing RTP properties. Moreover, visible light induces the isomerization of SPCOOH from MC to SP state, thereby terminating FRET between ME‐IPA and SPCOOH. Herein, we established a UV light‐triggered FRET‐enabled TDPC mechanism. This model strategically incorporates complementary photochromic and phosphorescent components within a polymer matrix to enable TS‐FRET. The resulting dual emission centers with distinct decay kinetics generate programmable TDPC behavior. This new strategy can be extended to other energy transfer systems to design and construct highly efficient RTP materials.

### 2.3 Dynamic Information Encryption Based on ME‐IPA‐SP_n_@PVA

**FIGURE 6:**
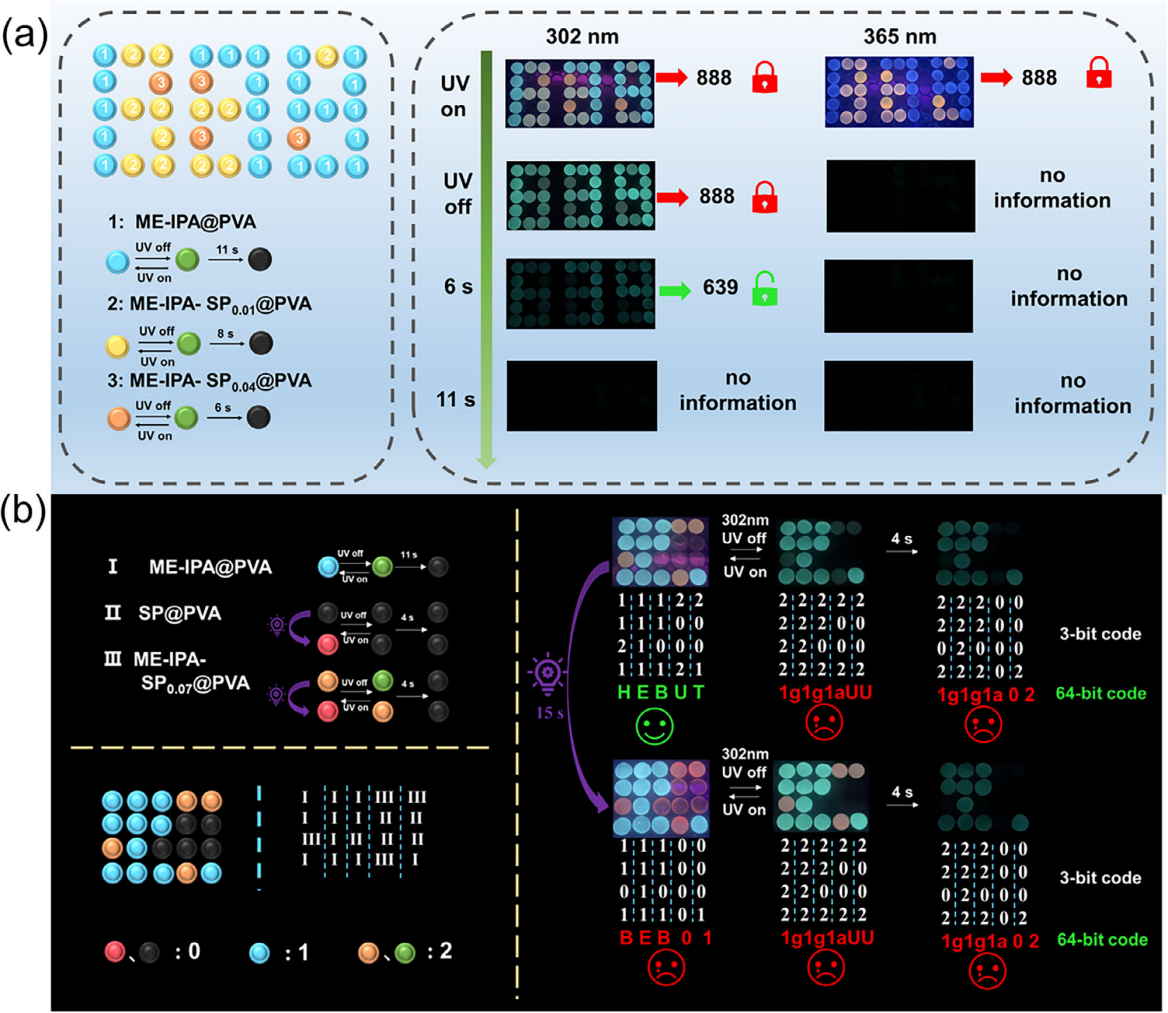
Information encryption and anti‐counterfeiting labels based on **ME‐IPA‐SP_n_@PVA** and **SP@PVA**.

The unique photo‐responsive RTP properties of **ME‐IPA‐SP_n_@PVA** film enabled multilevel information encryption and anti‐counterfeiting applications. As shown in Figure 6a, an encryption/decryption system was designed using **ME‐IPA@PVA**, **ME‐IPA‐SP_0.01_@PVA**, and **ME‐IPA‐SP_0.04_@PVA** films with different dynamic optical responses, exhibiting both time‐dependent and excitation‐wavelength‐dependent behaviors. Under 302 nm UV irradiation, the system displayed interference information “888” with varying fluorescence colors. Upon turning off the UV light, the following sequential transitions occurred: after 0.2 s, a green “888” appeared; at 6 s, it changed to correct information “639”; and ultimately at 11 s, all luminescence disappeared, effectively concealing the correct information. When exposed to 365 nm UV light, the system similarly showed an interference pattern “888” and invalid information.

To verify the feasibility of the material for more complex information storage and encryption security applications, we designed a time‐based multiple code encryption system using **ME‐IPA@PVA**, **SP@PVA** and **ME‐IPA‐SP_0.07_@PVA** films with different dynamic optical response characteristics. As shown in Figure 6b, **ME‐IPA@PVA** (I), **SP@PVA** (II) and **ME‐IPA‐SP_0.07_@PVA** (III) were arranged to form a 4 × 5 encryption matrix to hide the authentic and valid 64‐bit code information “H‐E‐B‐U‐T.” The complex 64‐bit code “H‐E‐B‐U‐T” can be encrypted into a 3‐digit code “1121‐1111‐1101‐2002‐2001” using the base conversion website (https://youkud.com/tool/jinzhi/index.html). Before UV irradiation, initial fluorescence colors of films I and III under 365 nm UV light excitation are blue and orange respectively, while film II shows no fluorescence emission, corresponding to digits “1,” “2” and “0” in the 3‐digit code. At this stage, the composed 3‐digit code “1121‐1111‐1101‐2002‐2001” can be converted to yield the correct 64‐bit code “H‐E‐B‐U‐T.” After removing the UV excitation source, films I and III switched to green phosphorescence, at which point the 3‐digit code changes to “2222‐2222‐2202‐2002‐2002,” yielding an invalid 64‐bit code “1g‐1g‐1a‐U‐U” upon conversion. After 4 s, when the green phosphorescence of film III disappeared, the 3‐digit code becomes “2202‐2222‐2202‐0000‐0002,” resulting in another invalid 64‐bit code “1g‐1g‐1a‐0‐2.” After 15 s of 365 nm UV light irradiation, under continued UV excitation, films II and III show significant fluorescence color changes while film I maintained its blue fluorescence. The 3‐digit code now reads “1101‐1111‐1101‐0000‐0001,” which converted to an interference 64‐bit code “B‐E‐B‐0‐1.” Upon removing the UV source again, films I and III switched to green and orange phosphorescence, respectively, while film II showed no luminescence, restoring the 64‐bit code to “1g‐1g‐1a‐U‐U.” After 4 s, the yellow phosphorescence of film III vanished, the invalid code “1g‐1g‐1a‐0‐2” reappeared. Additionally, exposing the decrypted information to visible light can conceal the authentic information, enabling multiple encryption and decryption cycles.

## 3 Conclusion

In summary, we successfully constructed intriguing photo‐responsive color‐tunable RTP polymer films with reversible photochromic and TDPC properties by doping the phosphorescence block ME‐IPA and the spiropyran‐based photochromic block SPCOOH into the PVA matrices. The reversible photoisomerization of SPCOOH component between its closed‐ring SP and open‐ring merocyanine MC states triggered by UV/Vis light enables controlled modulation of the material's luminescent color through selective activation of TS‐FRET between ME‐IPA (donor) and SPCOOH (acceptor). This energy transfer mechanism establishes dual phosphorescent emission pathways, generating TDPC characteristics through differential decay kinetics. Capitalizing on the dynamically reversible, high‐contrast, and rapidly responsive luminescent properties and excellent processability, various information encryption and anti‐counterfeiting labels were demonstrated.

## Conflicts of Interest

The authors declare no conflict of interest.

## Supporting information




**Supporting File**: advs74407‐sup‐0001‐SuppMat.docx.

## Data Availability

Research data are not shared.
