# Supplementary material for: Photo‐Responsive Room‐Temperature Phosphorescent Films Exhibiting Photochromism and Time‐Dependent Phosphorescent Colors for Multilevel Anti‐Counterfeiting
Source: Adv Sci (Weinh). 2026 Mar 3;13(24):e23121. doi: 10.1002/advs.202523121 (PMC13116334; doi:10.1002/advs.202523121)
Supplement: Supplementary file 1 — Supporting File: advs74407‐sup‐0001‐SuppMat.docx. [file ADVS-13-e23121-s001.docx]

**Supporting information**

Photo-responsive Room-Temperature Phosphorescent Films Exhibiting Photochromism and Time-Dependent Phosphorescent Colors for Multilevel Anti-Counterfeiting

Ziyi Bai, ^[a]^ ^‡^  Xinyi Xu, ^[a]^ ^‡^ Jinbo Fu, ^[a] ‡^  Di Zhao, ^[a]^ Yige Wang*^[a]^ and Huanrong Li*^[a]^

[a] Ziyi Bai, Xinyi Xu, Jinbo Fu, Di Zhao, Yige Wang and Huanrong Li
School of Chemical Engineering and Technology
Hebei University of Technology
GuangRong Dao 8, Hongqiao District, Tianjin 300130, P. R. China.
E-mail: wangyige@ hebut.edu.cn，lihuanrong@ hebut.edu.cn

^[‡]^ These authors contributed equally to this work.

* Corresponding author.

**2. EXPERIMENTAL SECTION**

**S1. Materials**

2,3,3-trimethylindole, 3-bromopropionic acid, 5-nitrosalicylaldehyde and polyvinyl alcohol (1795) were obtained from Aladdin (China). Melamine (ME) and isophthalic acid (IPA) were obtained from Macklin Reagent (Shanghai China).

**S2. Synthetic Procedures**

**(1) Synthesis of SPCOOH**

1.2 mL of 2,3,3-trimethylindole and 1.14 g of 3-bromopropionic acid were dissolved in 15 mL of anhydrous toluene and heated to reflux at 100 °C for 15 h. At the end of refluxing, the products were cooled to room temperature, and a purple-black solid was obtained by filtration. The product was washed three times with anhydrous toluene and dissolved in dichloromethane, followed by slow addition to ethyl acetate to precipitate an orange-red solid. After filtration and drying at 50 °C for 24 h, the intermediate C-TMI-Br was obtained. Yield: 53.33%.

0.20 g of intermediate C-TMI-Br and 0.13 g of 5-nitrosalicylaldehyde were dissolved in 10 ml of anhydrous ethanol to which 0.15 ml of triethylamine was added and heated to reflux at 90°C for 10 h. At the end of refluxing, the products were cooled down to room temperature, filtered and dried, and then purified on a silica column to obtain a product, denoted as SPCOOH. Yield: 60.94%. (The ratio of the eluents was petroleum ether: ethyl acetate = 4:6)

**(2) Synthesis of ME-IPA**

126 mg of ME was added to 30 mL of distilled water, stirred at 95°C until completely dissolved, and when the temperature was lowered to 70°C, 166 mg of IPA was added and reacted for 3 h to obtained ME-IPA.^[32]^

**(3) Synthesis of** **ME-IPA-SP_n_@PVA film**

126 mg of ME was added to 30 mL of distilled water and stirred at 95 °C until complete dissolution, followed by the addition of 1.50 g of PVA, and after 1 h of reaction the temperature was lowered to 70°C, and 166 mg of IPA dispersed in 15 mL of distilled water was added under vigorous stirring, and the reaction was continued for 3 h to obtain the precursor solution, denoted as ME-IPA@PVA. 2 mL of ME-IPA@PVA solution was taken and 240 μL of SPCOOH-EtOH solution (c=0.2 mol/L) was added dropwise under vigorous stirring, and react at room temperature for 3 h. Then the above solution was poured into a Petri dish and dried on a heating plate at 45°C to form a film, which was recorded as ME-IPA-SP_0.04_@PVA. In addition, a series of precursors were synthesized using the same procedure by adjusting the volume of SPCOOH-EtOH solution, a series of films were synthesized using the same procedure (n=0.01, 0.07).

**S3. Characterizations**

FT-IR spectra were measured on the Bruker TENSOR 27 spectrometer instrument in the range from 400 to 4000 cm^−1^ (16 scans, resolution: 4 cm^−1^). The UV-Vis absorption spectra and transmittance spectra were tested on an Agilent Cary 100 UV-Vis spectrometer at room temperature. The luminescence spectra and decay time tests were measured on Edinburgh Instruments FS920P spectrometer. Scanning electron microscopy (SEM) and energy-dispersive X-ray spectroscopy (EDS) images were obtained by a TESCAN MIRA LMS. The X-ray diffraction (XRD) patterns were carried out on Bruker M18U spectrometer.


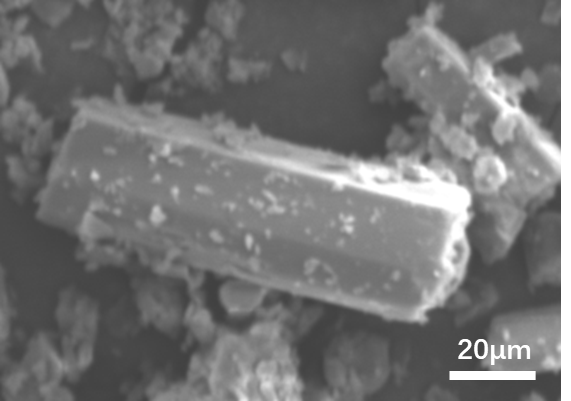


Figure S1. SEM images of ME-IPA


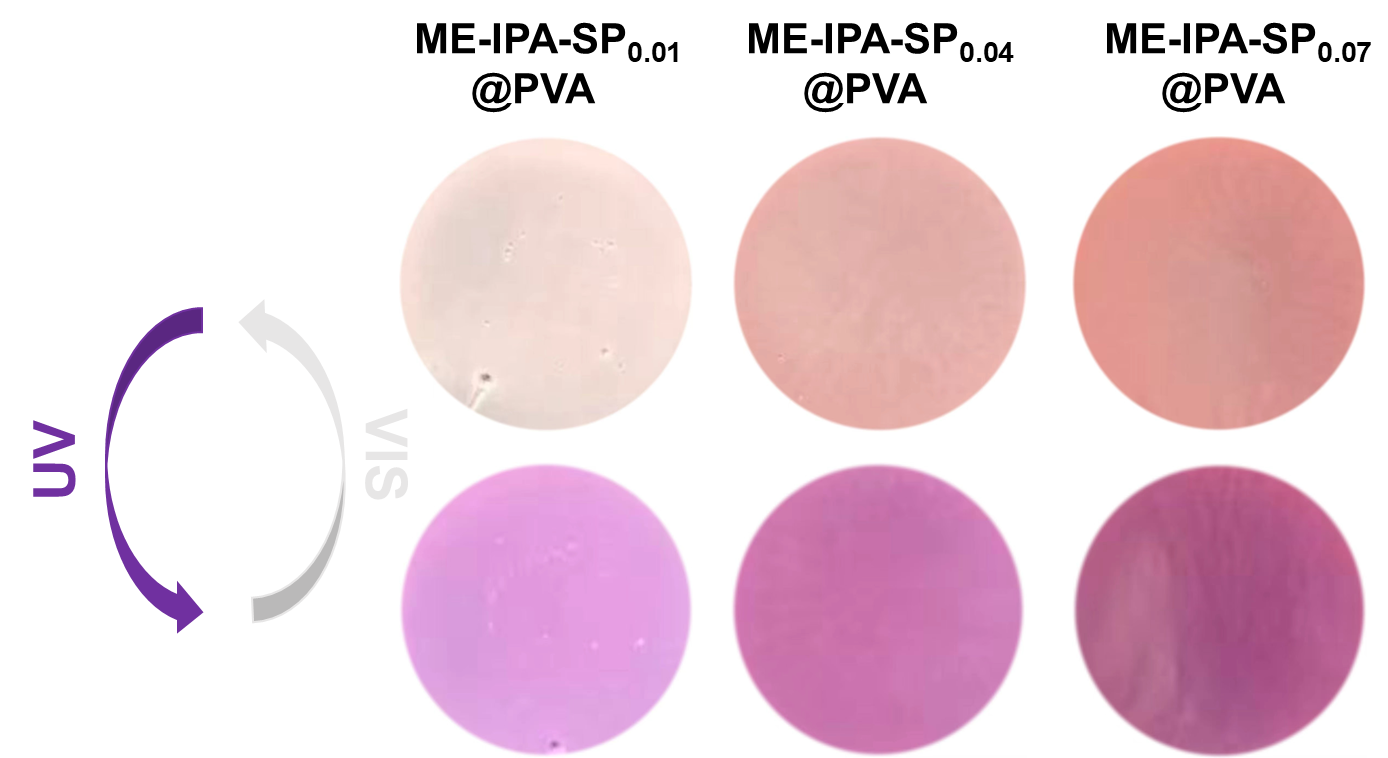


Figure S2. Photographs of ME-IPA-SP_n_@PVA upon daylight.

Figure S3. UV-vis absorption spectrum of ME-IPA-SP_0.04_@PVA under 365 nm UV irradiation and visible light irradiation.


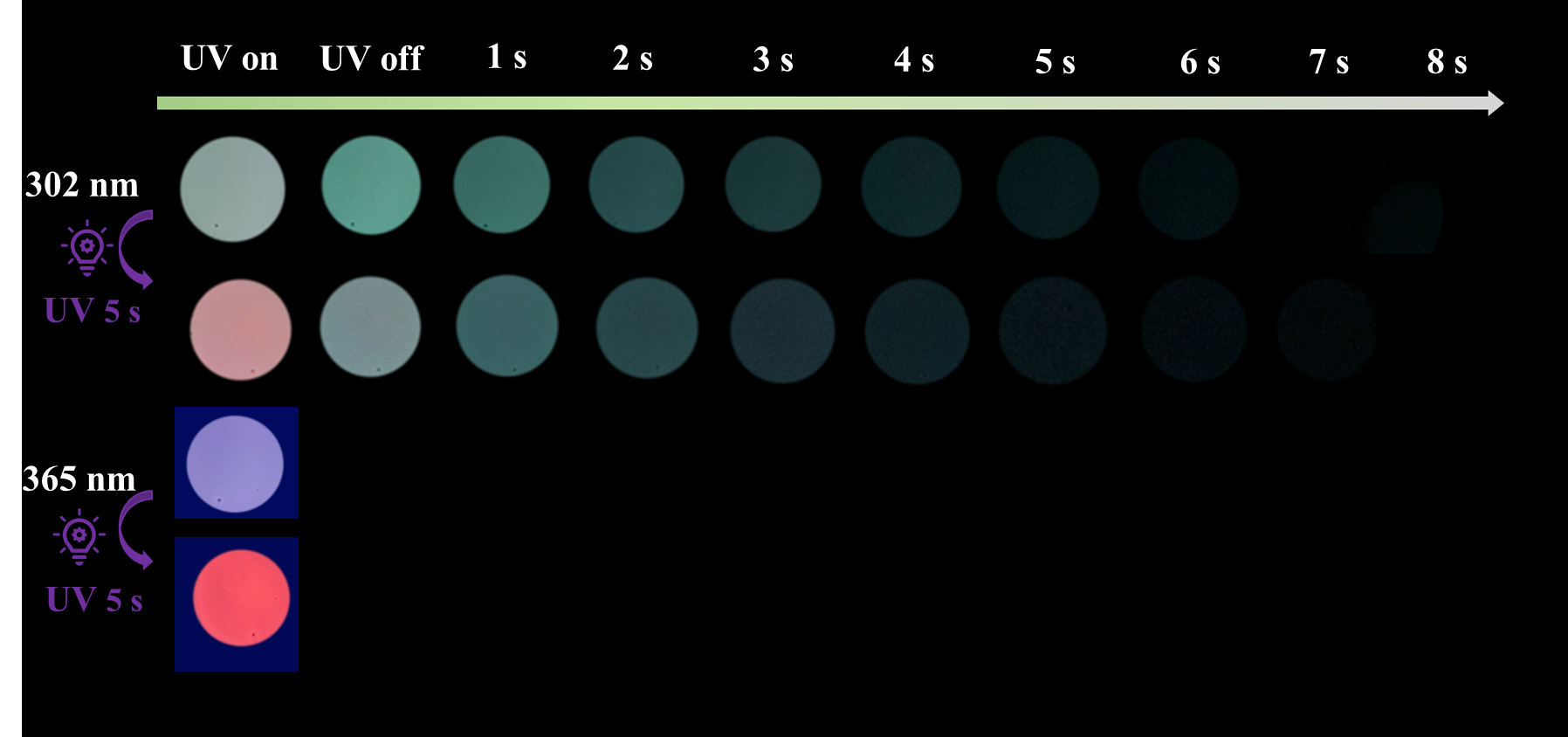


Figure S4. Photographs of ME-IPA-SP_0.01_@PVA and ME-IPA-MC_0.01_@PVA upon daylight, 302 and 365 nm UVon and UV off conditions


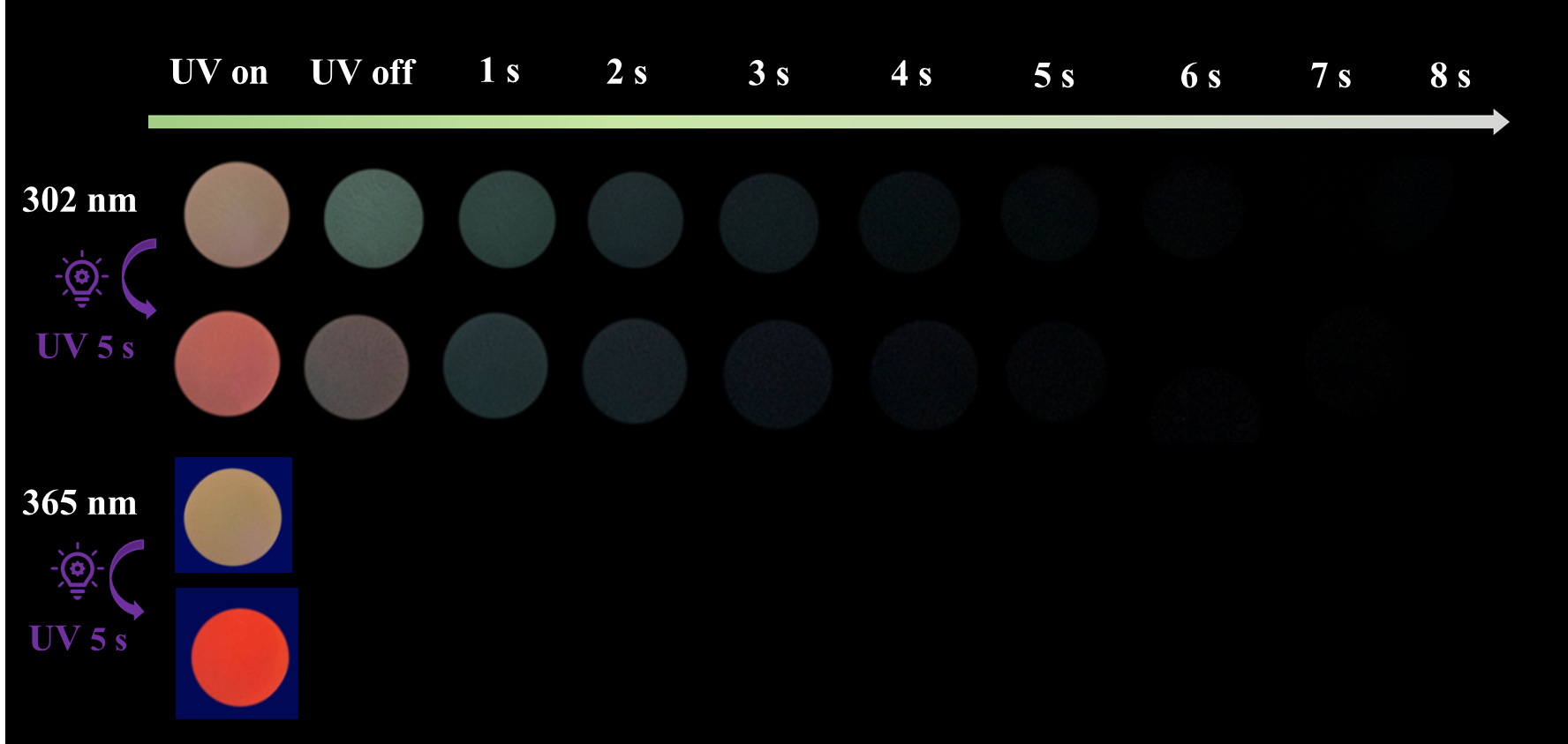


Figure S5. Photographs of ME-IPA-SP_0.07_@PVA and ME-IPA-MC_0.07_@PVA upon daylight, 302 and 365 nm UVon and UV off conditions


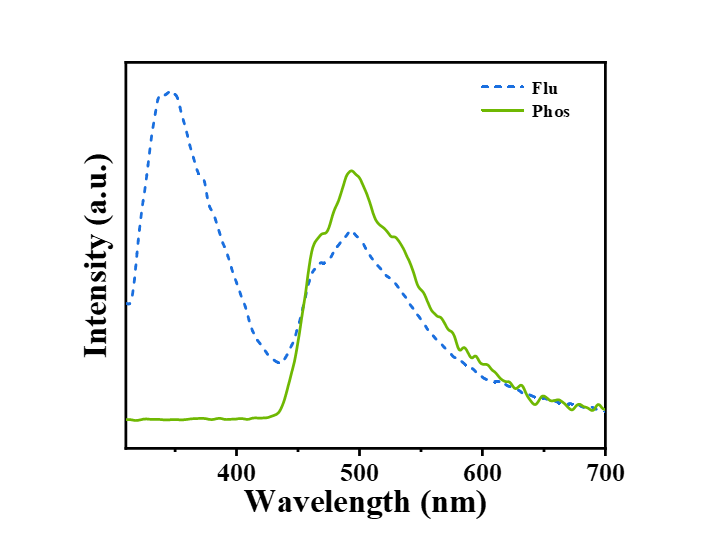


Figure S6. (a) Steady-state photoluminescence (PL, blue dashed line) and phosphorescence (geen solid line) spectra of ME-IPA under 300 nm excitation


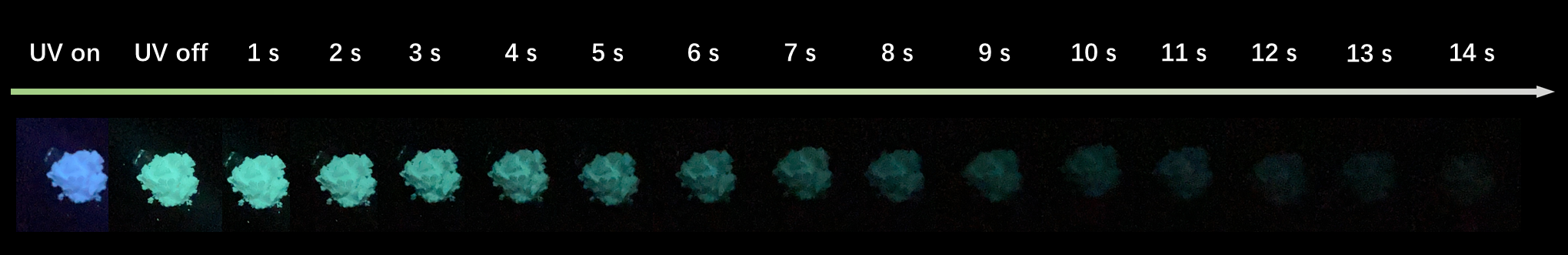


Figure S7. Photographs of ME-IPA taken before and after turn-off of the 302 nm excitation in the scale from 0 to 14 s

Figure S8. Phosphorescence decay curves of ME-IPA-SP_n_@PVA films (λ_ex_=300 nm, λ_em_=625, 655nm)


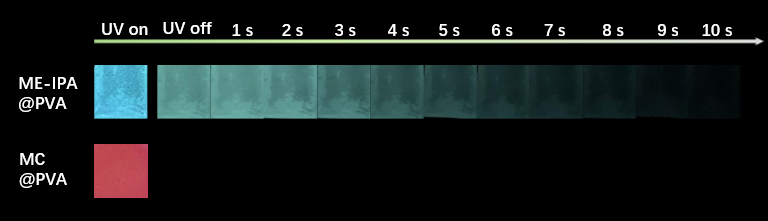


Figure S9. Photographs of luminescence behaviors for ME-IPA@PVA and MC@PVA upon 302 nm UVon and UV off


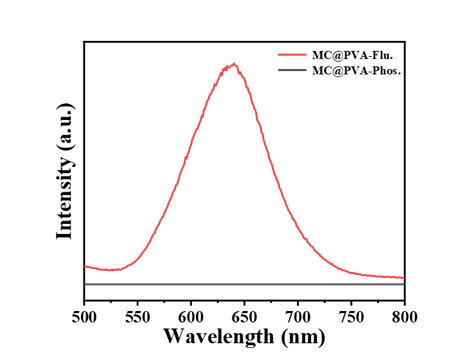


Figure S10. Fluorescent and phosphorescent spectra of MC@PVA monitored in the same slit model (𝜆𝑒𝑥= 300 nm).

Figure S11. Fluorescent decay curves of MC@PVA at 645 nm (λ_ex_=300 nm).


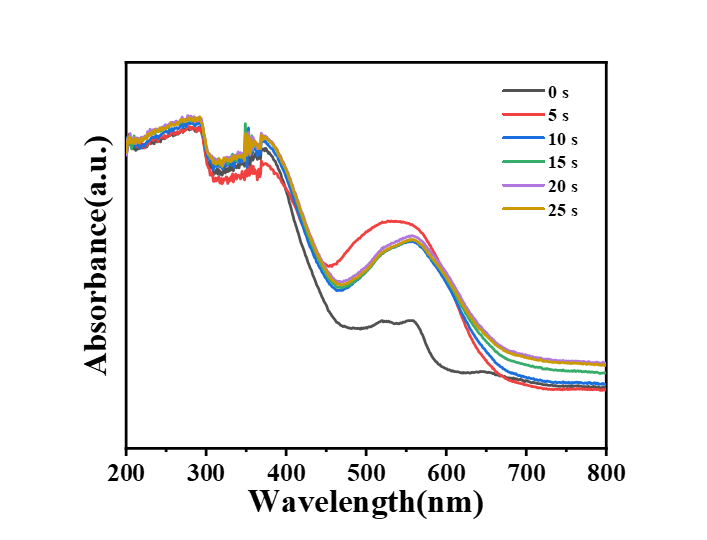


Figure S12. UV-vis absorption spectrum of ME-IPA-SP_0.04_@PVA under continuous exposure to 365 nm UV light.

Figure S13. Phosphorescence decay curves of ME-IPA-SP_0.04_@PVA under continuous exposure to 365 nm UV light (λ_ex_=300 nm, λ_em_=493).

Table S1. Relationship between UV irradiation time, SP→MC photoisomerization conversion, and TS-FRET efficiency for ME‑IPA‑SP_0.04_@PVA.

| 365 nm UV irradiation time /s | 0 | 5 | 10 | 15 | 20 | 25 |
| --- | --- | --- | --- | --- | --- | --- |
| UV absorption value (558 nm) /a.u. | 0.54 | 0.61 | 0.60 | 0.60 | 0.60 | 0.60 |
| Conversion rate (SP→MC) /% | / | 13.0 | 11.1 | 11.1 | 11.1 | 11.1 |
| Lifetime (493 nm) /s | / | 1.07 | 1.23 | 1.27 | 1.25 | 1.22 |
| *η_ET_* /% | / | 31.4 | 21.2 | 18.6 | 19.9 | 21.8 |


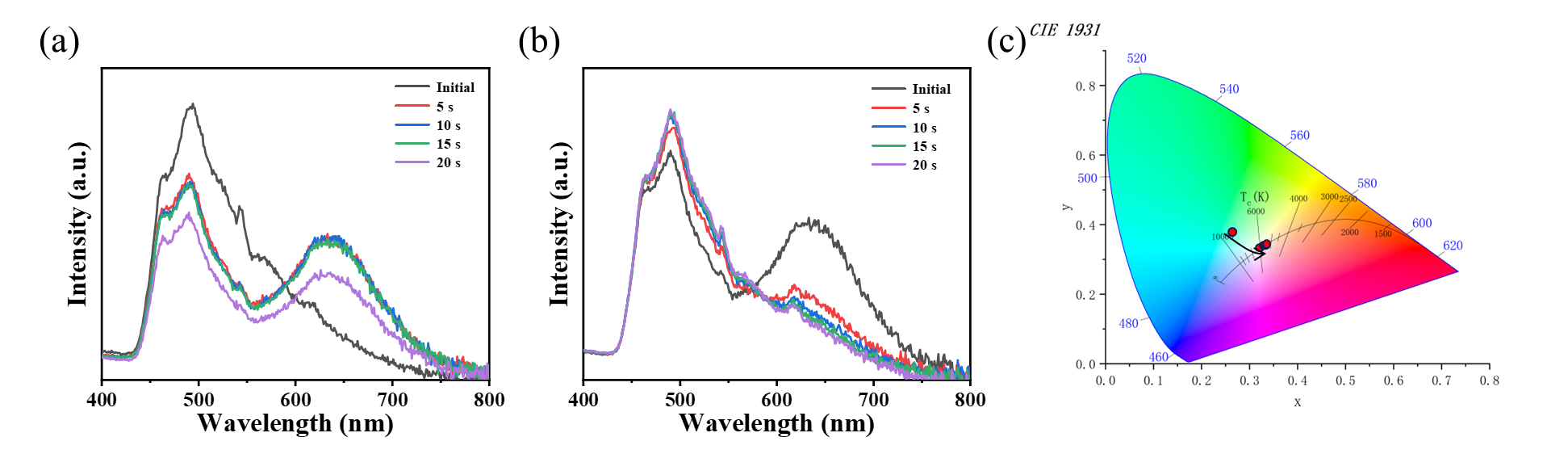


Figure S14. Time-dependent phosphorescence spectra of ME-IPA-SP_0.01_@PVA film under (a) 365 nm UV irradiation and the visible light irradiation; (c) CIE coordinates of afterglow emission for ME-IPA-SP_0.01_@PVA film under the 365 nm UV irradiation


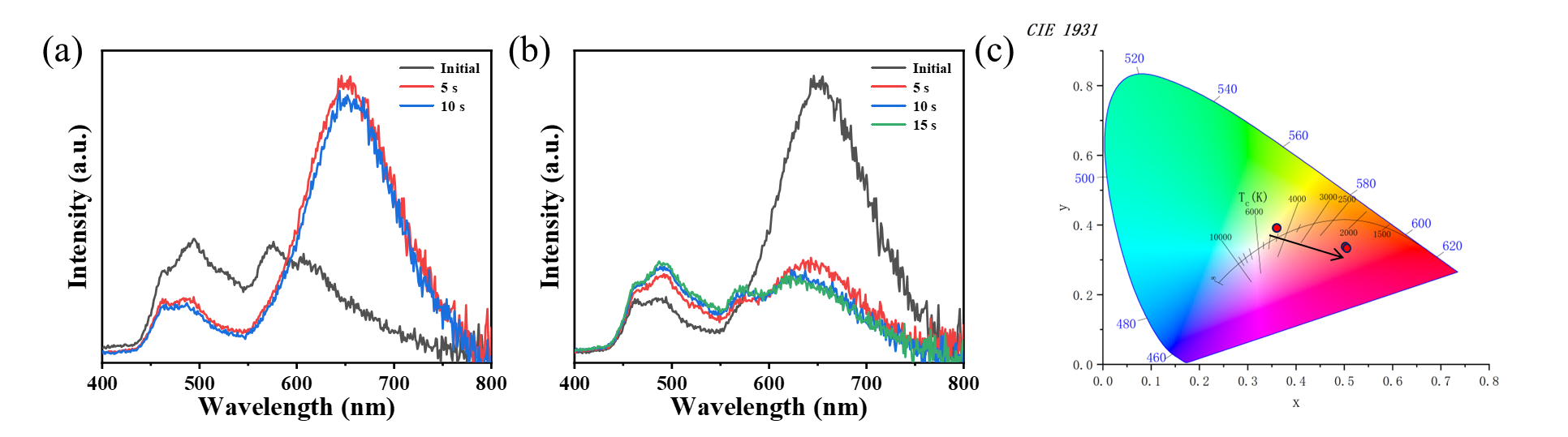


Figure S15. Time-dependent phosphorescence spectra of ME-IPA-SP_0.07_@PVA film under (a) 365 nm UV irradiation and the visible light irradiation; (c) CIE coordinates of afterglow emission for ME-IPA-SP_0.07_@PVA film under the 365 nm UV irradiation


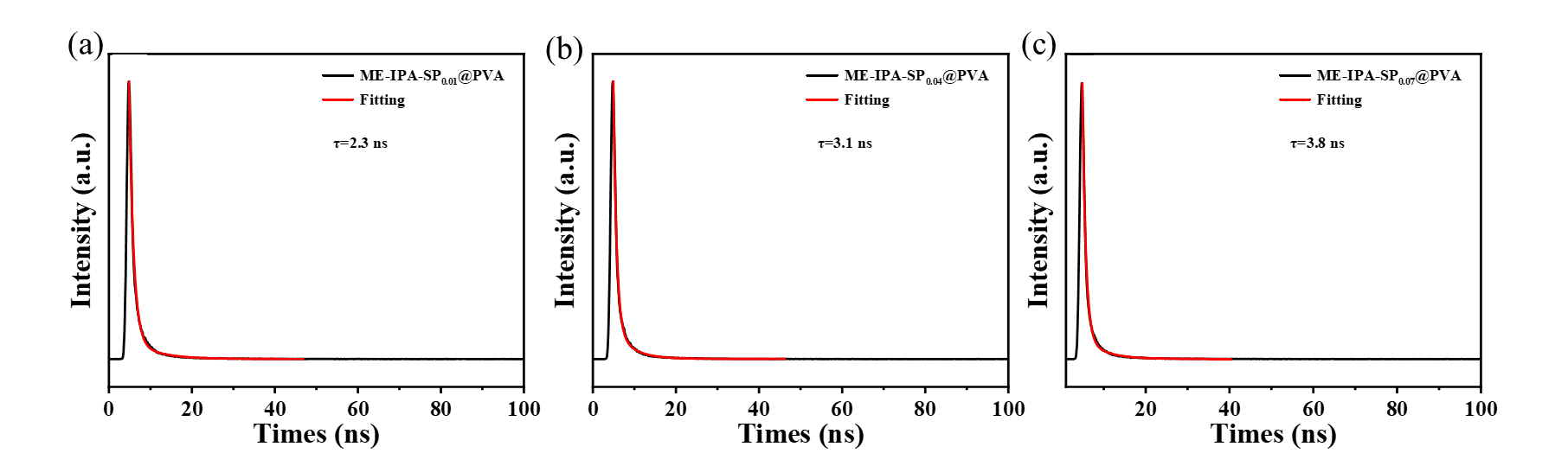


Figure S16. Singlet lifetime of ME-IPA-SP_n_@PVA film (a) λ_ex_=300 nm, λ_em_=625 nm; (b) λ_ex_=300 nm, λ_em_=655 nm; (c) λ_ex_=300 nm, λ_em_=655 nm.
